# Modulation of Angiogenic and Inflammatory Response in Glioblastoma by Hypoxia

**DOI:** 10.1371/journal.pone.0005947

**Published:** 2009-06-17

**Authors:** Anastasia Murat, Eugenia Migliavacca, S. Farzana Hussain, Amy B. Heimberger, Isabelle Desbaillets, Marie-France Hamou, Curzio Rüegg, Roger Stupp, Mauro Delorenzi, Monika E. Hegi

**Affiliations:** 1 Laboratory of Brain Tumor Biology and Genetics, University Hospital Lausanne (CHUV) and University of Lausanne, Lausanne, Switzerland; 2 Department of Neurosurgery, University Hospital Lausanne (CHUV) and University of Lausanne, Lausanne, Switzerland; 3 National Center of Competence in Research (NCCR) Molecular Oncology, ISREC, School of Life Sciences, EPFL, Lausanne, Switzerland; 4 Swiss Institute for Bioinformatics, Lausanne, Switzerland; 5 Department of Neurosurgery, The University of Texas M. D. Anderson Cancer Center, Houston, Texas, United States of America; 6 Division of Experimental Oncology, Centre Pluridisciplinaire d'Oncologie, CHUV and University of Lausanne, Lausanne, Switzerland; Institute of Cancer Research, United Kingdom

## Abstract

Glioblastoma are rapidly proliferating brain tumors in which hypoxia is readily recognizable, as indicated by focal or extensive necrosis and vascular proliferation, two independent diagnostic criteria for glioblastoma. Gene expression profiling of glioblastoma revealed a gene expression signature associated with hypoxia-regulated genes. The correlated gene set emerging from unsupervised analysis comprised known hypoxia-inducible genes involved in angiogenesis and inflammation such as *VEGF* and *BIRC3*, respectively. The relationship between hypoxia-modulated angiogenic genes and inflammatory genes was associated with outcome in our cohort of glioblastoma patients treated within prospective clinical trials of combined chemoradiotherapy. The hypoxia regulation of several new genes comprised in this cluster including *ZNF395, TNFAIP3, and TREM1* was experimentally confirmed in glioma cell lines and primary monocytes exposed to hypoxia *in vitro*. Interestingly, the cluster seems to characterize differential response of tumor cells, stromal cells and the macrophage/microglia compartment to hypoxic conditions. Most genes classically associated with the inflammatory compartment are part of the NF-kappaB signaling pathway including *TNFAIP3* and *BIRC3* that have been shown to be involved in resistance to chemotherapy.

Our results associate hypoxia-driven tumor response with inflammation in glioblastoma, hence underlining the importance of tumor-host interaction involving the inflammatory compartment.

## Introduction

Maintenance of oxygen homeostasis is critical for cell survival. Hypoxia is a common condition in cancer tissue due to rapid tumor growth, accompanied by inadequate angiogenesis with formation of structurally aberrant, leaky blood vessels with poor blood flow and formation of edema. In fact, such aberrant vascular proliferation characterized by glomeruloid and garland-like patterns are a hallmark of glioblastoma [1], the most malignant primary brain tumor. Cancer cells undergo adaptive changes and are selected for genetic alterations that allow them to survive and proliferate in a hypoxic environment. Hypoxia-regulated genes, mediating adaptive physiologic changes, include genes regulating the glycolytic pathway and blood-vessel formation, and genes encoding chemotactic molecules such as CCL2, IL8 and VEGF [2]. In cancer, such changes are associated with recruitment of macrophages along a hypoxia-mediated chemotactic gradient. Macrophages recruited to hypoxic sites exert a tumor-promoting effect through the expression of genes with mitogenic, angiogenic, and migration/invasion stimulating properties, such as *VEGF, EGF*, or *HGF* [3], [4]. The relative contribution of hypoxia-induced genes expressed by tumor cells or macrophages to tumor progression is unknown. Tumor hypoxia is associated with aggressive tumor behavior and worse outcome in many cancers and its role in driving tumor malignancy and resistance to therapy is receiving increased attention.

The key mediator of the molecular responses to hypoxia is the hypoxia-inducible factor-1 (HIF-1), a heterodimeric transcription factor consisting of an α and a β subunits. In the presence of oxygen the HIF-1α subunit is hydroxylated, and upon ubiquitination is targeted to the proteasome for degradation. Under hypoxic conditions, however, HIF-1α hydroxylation and degradation no longer occur, since the hydroxylation reaction requires oxygen. Stabilized, non-hydroxylated HIF-1α translocates to the nucleus and binds to the hypoxia-response element (HRE) thereby activating expression of numerous hypoxia-responsive genes [5]. Hypoxia-inducible pathways regulate several biological processes, including angiogenesis, cell proliferation, metabolism, survival and apoptosis, immortalization, and migration. Besides hypoxia, HIF-1 can also be activated by growth factor receptors and oncogenic signaling pathways.

Using gene expression profiling of human gliomas, we have previously identified angiogenesis/response to hypoxia as one of the most discriminating features between malignancy grades of astrocytic glioma [6]. Accordingly, a hypoxia-induced gene expression signature is a feature of glioblastoma expression profiles [7], [8] which has been reported to classify gliomas into prognostic groups in some datasets [6], [9].

The aim of the present study was to investigate biological and potential clinical implications of a hypoxia-related gene signature emerging from our glioblastoma gene expression data set [7]. All patients were enrolled in a prospective clinical trial of combined chemoradiotherapy for newly diagnosed glioblastoma [10].

## Results

### Hypoxia-inducible genes related to angiogenesis and inflammation

**Figure 1:**
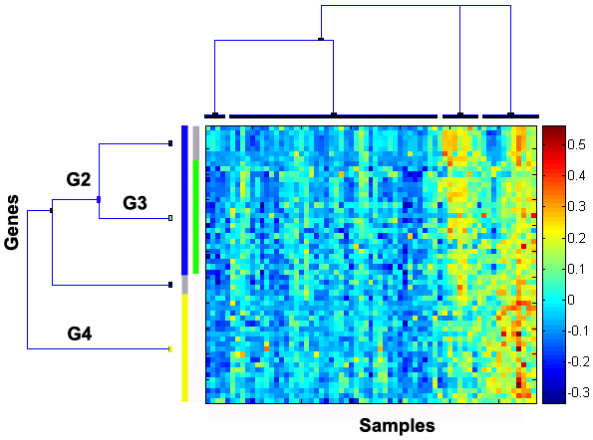
The hypoxia gene cluster. Gene dendrogram and heat map of the hypoxia cluster, showing two main gene groups; G2, enriched for inflammatory genes (yellow bar) and G4, containing angiogenesis-related genes (blue bar). G3 (green bar) is a subcluster of G4 (grey bars mark genes not organized in stable subclusters as defined by CTWC), see Table 1 for detailed cluster information.

**Figure 2:**
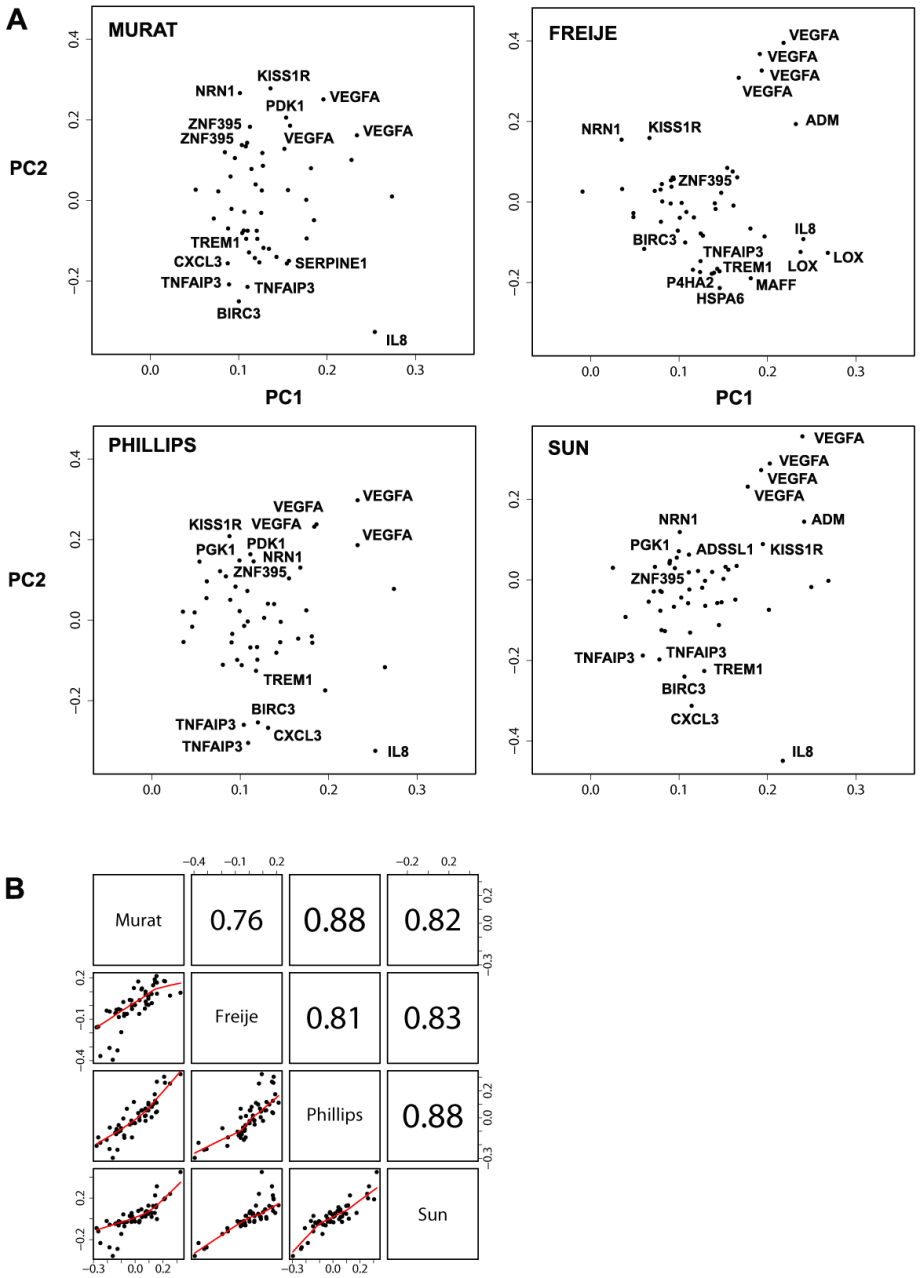
Components of the hypoxia gene cluster. *A*, Loading plots, representing the coefficients of the linear combination of the 52 common probe-sets of the hypoxia cluster used to define the first two Principal Components (PC) (Table 1), are shown for our data-set and 3 published data-sets [8], [9], [12]. In all data-sets genes with the highest positive coefficients in the linear combination defining the 2^nd^ PC include *VEGF, ZNF395* and *KISS1R*. Genes with the highest negative contribution to the 2^nd^ PC comprise *TREM1, TNFAIP3, BIRC3* and *IL8*. Probe-sets with the most extreme contributions to the 2^nd^ PC in all data-sets are labeled. *B,* Pair wise scatter plots and Pearson correlations of the loadings of the 52 probe-sets in the 2^nd^ PC across our data-set (n = 69), and the external data-sets Freije (n = 48), Phillips (n = 54), and Sun (n = 71).

Unsupervised analysis of our gene expression data-set derived from 80 glioblastoma and 4 non-tumoral brain tissues identified stable gene clusters that were associated with known biological processes, including a cluster characterized by hypoxia-induced genes as shown in Figure 1, Table 1. The hypoxia cluster drew our attention, when we found that the second principle component (PC) of this cluster was strongly associated with survival (p = 0.0015; HR, 1.73; 95% CI, 1.23 to 2.43; multivariate analysis including age, treatment, *MGMT*-methylation status [a predictive factor for benefit from temozolomide treatment [11]]), while the 1^st^ PC, as well as the mean expression of the cluster, had no prognostic value in our cohort of patients. Subsequent investigation of the loadings (coefficients of the genes) in the PCs of the hypoxia cluster revealed that the 2^nd^ PC was mainly defined by two groups of genes: the first characterized by high negative coefficients and comprising *IL8, TREM1, SERPINE1, BIRC3*, and *TNFAIP3;* the second characterized by high positive coefficients and consisting of genes such as *VEGF, ADM, ZNF395*, and *KISS1R* (Fig. 2A). The first group is enriched for inflammation-related genes, while the second group consists more of universal hypoxia-regulated genes, including pro-angiogenic factors, such as *VEGF* and *ADM*, in accordance with the gene dendrogram of the hypoxia cluster (Fig. 1, Table 1). Next we tried to identify biological features, characterized by gene expression, that might be correlated with the 2^nd^ PC. In our data-set we identified a list of genes enriched in inflammatory/innate immune response genes (e.g. *S100A8*, *S100A9*, *ITGB2, TLR1*) that were anti-correlated with the 2^nd^ PC, and a list of positively correlated genes comprising signal transduction-related genes. Samples with lower 2^nd^ PC have higher expression of inflammatory genes and vice versa. In accordance, our previously published immune response-related cluster (**G24**) displayed a similar correlation (−0.54, Pearson correlation of mean expression) with the 2^nd^ PC. The top anti-correlated genes of the 2^nd^ PC (cut-off at −0.5 correlation) that passed the initial variation filter, were almost exclusively clustered in **G24** (33/46 genes, Table S1).

**Table 1:** 1Probesets of the Hypoxia Gene Cluster.

Probe-set	1^st^ PC 2Coeficients	2^nd^ PC 2Coeficients	Gene Symbol	3Cluster	4Hypoxia Regulation
203438_at	0.10	0.11	STC2	G4	4
218149_s_at	0.11	0.18	ZNF395	G4	this work
221123_x_at	0.11	0.13	ZNF395	G4	this work
223216_x_at	0.11	0.14	FBXO16 /// ZNF395	G4	this work
232693_s_at	0.10	0.14	FBXO16 /// ZNF395	G4	this work
242517_at	0.14	0.28	KISS1R	G4	this work
51553392_at	–	–	FLJ25818	G4	
51554452_a_at	–	–	HIG2	G3 G4	4
201170_s_at	0.11	−0.07	BHLHB2	G3 G4	4
205525_at	0.05	0.03	CALD1	G3 G4	
210512_s_at	0.20	0.25	VEGFA	G3 G4	4
210513_s_at	0.15	0.13	VEGFA	G3 G4	4
211527_x_at	0.23	0.16	VEGFA	G3 G4	4
212171_x_at	0.16	0.19	VEGFA	G3 G4	4
218507_at	0.18	0.08	HIG2	G3 G4	4
219410_at	0.13	0.12	TMEM45A	G3 G4	
222646_s_at	0.18	0.00	ERO1L	G3 G4	4
223484_at	0.11	−0.10	C15orf48	G3 G4	
224797_at	0.12	0.04	ARRDC3	G3 G4	
226452_at	0.15	0.21	PDK1	G3 G4	4
226325_at	0.08	0.12	ADSSL1	G3 G4	
218625_at	0.10	0.27	NRN1	G3 G4	4
209122_at	0.18	−0.09	ADFP	G3 G4	4
207850_at	0.09	−0.16	CXCL3	G3 G4	
203282_at	0.09	0.06	GBE1	G3 G4	4
202912_at	0.23	0.10	ADM	G3 G4	4
202497_x_at	0.13	−0.12	SLC2A3	G3 G4	4
201313_at	0.08	0.02	ENO2	G3 G4	4
200737_at	0.11	0.08	PGK1	G3 G4	4
202499_s_at	0.16	−0.15	SLC2A3		4
36711_at	0.14	−0.14	MAFF		4
222088_s_at	0.12	−0.08	SLC2A14 /// SLC2A3		4
202859_x_at	0.25	−0.33	IL8		4
202934_at	0.13	0.09	HK2	G2	4
203108_at	0.11	−0.08	GPRC5A	G2	4
204298_s_at	0.19	−0.05	LOX	G2	4
204508_s_at	0.11	−0.03	CA12	G2	4
210538_s_at	0.10	−0.25	BIRC3	G2	4
212110_at	0.13	−0.12	SLC39A14	G2	
215446_s_at	0.27	0.01	LOX	G2	4
219434_at	0.12	−0.14	TREM1	G2	this work, 4
221009_s_at	0.16	0.03	ANGPTL4	G2	4
222939_s_at	0.11	−0.13	SLC16A10	G2	4
236220_at	0.07	−0.04	–	G2	
230746_s_at	0.10	−0.08	STC1	G2	4
226722_at	0.09	−0.07	FAM20C	G2	
213418_at	0.12	−0.15	HSPA6	G2	
204595_s_at	0.12	−0.10	STC1	G2	4
202733_at	0.09	−0.02	P4HA2	G2	4
202644_s_at	0.09	−0.21	TNFAIP3	G2	this work
202643_s_at	0.11	−0.21	TNFAIP3	G2	this work
202627_s_at	0.15	−0.16	SERPINE1	G2	4
201169_s_at	0.13	−0.03	BHLHB2	G2	4
200632_s_at	0.13	0.02	NDRG1	G2	4

1 54 probe-sets of gene cluster **G84** as described [7] ordered according to the CWTC gene dendrogram of the cluster shown in Fig. 1.

2 Coefficients for the probesets defining the 1^st^ and 2^nd^ PC in the hypoxia cluster.

3 G2 and G4 are stable gene subclusters of the hypoxia cluster; **G3** is a stable subcluster of **G4;** see Fig. 1.

4 Published evidence for hypoxia regulation, references in Table S2.

5 Probe-sets not part of 52 probe-sets common to the 3 external data-sets [8], [9], [12].

To investigate if the two directions that account for the largest variance in the hypoxia cluster were consistent in different glioblastoma cohorts, we analyzed three external datasets [8], [9], [12] including only samples labeled as glioblastoma (WHO grade IV) from initial surgery (no other prior therapy). We performed PC analysis using as descriptive variables 52 probe-sets from our hypoxia cluster that were common to all datasets (Table 1). The loading plots of all datasets displayed a similar influence of the genes to the first two PCs: the group of genes containing *IL8*, *TREM1, SERPINE1, BIRC3*, and *TNFAIP3* were separated from the group containing *VEGF, ADM, ZNF395*, and *KISS1R* in the 2^nd^ PC (Fig. 2A). In particular, most coefficients of genes in the first group are negative and most coefficients of genes in the second group are positive in all the four datasets. Hence, there is a reproducible pattern in which a consistent component of tumor variability is described by the differential expression of these two groups of genes.

**Figure 3:**
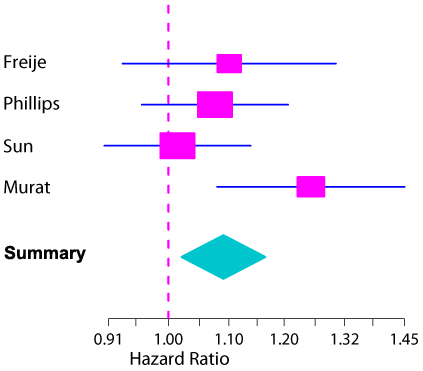
Meta-analysis using four gene expression data-sets of glioblastoma. The Forest plot visualizes the prognostic value of the meta analysis using the 2^nd^ PC of the hypoxia cluster in a Cox model of four glioblastoma data-sets (n = 242, p = 0.010, HR, 1.09, 95% CI, 1.02 to 1.16) [7], [8], [9], [12]. When combining the three external data-sets formal statistical significance (alpha level of 5%) was not reached (HR = 1.06 95% CI: 0.98, 1.14, p-value: 0.15).

We observed that the loading vectors for the four datasets were highly correlated, both for the 1^st^ and the 2^nd^ PC, while there is a dramatic drop of the correlations for the 3^rd^ PC loadings. The pair-wise correlations between the 2^nd^ PC loadings of the four datasets range from 0.76 to 0.88 (Fig. 2B). To examine the association of the 2^nd^ PC with survival, a combined analysis of the four cohorts was performed using a Cox proportional hazards model with stratification by study. This model is in agreement with a potential prognostic value of the 2^nd^ PC (n = 242, p = 0.010, HR, 1.09, 95% CI, 1.02 to 1.16) (Fig. 3). However, if we consider a Cox model combining just the three external studies, the 2^nd^ PC does not reach statistical significance at the conventional 5% significance level (HR = 1.06 95% CI: 0.98, 1.14, p-value: 0.15). It is of note that the patients in these datasets are more heterogeneous since, unlike our study, they have not been collected prospectively and were not treated uniformly.

The inflammation-related gene set (negative coefficients in the linear combination defining the 2^nd^ PC) contains *CXCL3, TNFAIP3* and *BIRC3*, three NF-κB down-stream target genes [13], [14] known to be expressed by macrophages [15], [16]. Other members of this group include *ADFP* and *SLC2A3* (*GLUT3*). Both genes, together with *IL8* and *SERPINE1*, have been reported to be highly expressed in macrophage foam cells [17], [18], [19], [20], in accordance with the presence of phagocytic cells in necrotic tissues. Thus, the re-grouping of these genes as observed in the 2^nd^ PC loading is consistent with their reported expression by macrophages.

In contrast, the genes in the second group (positive coefficients in the linear combination defining the 2^nd^ PC) may be expressed by any cell type and reflect essential adaptive responses to hypoxia. The genes in this group comprise angiogenic factors such as *VEGF*, *ADM*, PDK-1, encoding an enzyme of the glycolytic pathway active in hypoxic tumors [21], and survival factors, such as *NTN-1* (Neuritin) [22], which is also expressed by microendothelial cells in perinecrotic areas [23].

Taken together, the hypoxia cluster seems to capture the hypoxia-induced genes in the tumor as a whole, while the loading plot of the 2nd PC provides some information reflecting a more specific patho-physiological context associated with the presence of tumor-infiltrating monocytes/macrophages. These cells may be attracted by the tumors' hypoxic areas and necrosis, and may subsequently contribute to the inflammatory signature (enriched on the negative side of the loading plot) within the cluster of hypoxia-inducible genes. Thus the two PCs of this cluster seem to mirror differential response of glioblastoma to hypoxic conditions: a basic angiogenic response of the tumor cell compartment on one hand, and a more specific inflammatory response of macrophages /microglia on the other.

### Upregulation of inflammation-related genes in microglia/macrophages isolated from glioblastoma tissue

To investigate differential gene expression in the distinct tumor compartments, we performed gene expression profiling of paired samples of glioblastoma tissue and the respective glioma-infiltrating microglia/macrophage (GIM) cell fraction. The GIM cell fraction was isolated by a modified Percoll-gradient that minimizes artificial microglia/macrophage activation and enriches CD11b+/CD11c+/CD45+ cells as previously described [24]. In accordance, gene expression profiles exhibited at least two-fold increased RNA expression of the microglia/macrophage markers CD11b, CD11c, and CD45 as compared to the level of expression in the tumor tissue (Table 2). Similarly, the GIM fraction also expressed at least four-fold increased RNA levels of Fc receptor genes II and III (CD32 and CD16) and CD14. The genes with negative coefficients in the 2^nd^ PC (inflammation related, hypoxia-regulated genes) exerted an increased expression in the GIMs as compared to the respective tumor tissue, suggesting enrichment of the cells expressing these genes in the GIM fraction. Of the genes belonging to the second group (positive coefficients in the 2^nd^ PC), most displayed a lower level of expression in the GIMs as compared to the unsorted tumor bulk (Table 2). However, these gene expression levels are merely indicative of expression by microglia/macrophages compared to the tumor bulk, since the isolation procedure may have introduced some artifacts.

**Table 2:** Differential Expression of Microglial Markers and Selected Genes from the 2nd PC.

Probe-set ID	Name		Fold-change log_2_[Migroglia/GBM]
**Selected genes present in 2^nd^ PC**			
* Genes with negative contribution in 2^nd^ PC*			
202859_x_at	IL8	6.11	
202644_s_at	TNFAIP3	1.52	
219434_at	TREM1	1.58	
209122_at	ADFP	1.08	
210538_s_at	BIRC3	−0.10	
202627_s_at	SERPINE1	0.71	
207850_at	CXCL3	0.53	
* Genes with positive contribution in 2^nd^ PC*			
218625_at	NRN1	−6.19	
226452_at	PDK1	−1.31	
210512_s_at	VEGFA	−0.82	
242517_at	KISS1R	−0.36	
218149_s_at	ZNF395	0.06	
200737_at	PGK1	−1.69	
**Microglial Markers**			
205785_at	ITGAM (CD11b)	1.56	
210184_at	ITGAX (CD11c)	2.39	
212588_at	PTPRC (CD45)	2.57	
203561_at	FCGR2A (CD32)	3.43	
204006_s_at	FCGR3A /// FCGR3B (CD16)	4.01	
204007_at	FCGR3B (CD16b)	4.13	
201743_at	CD14	2.00	

### Novel hypoxia-induced genes in the expression profiles of glioblastoma patients

More than two-thirds of the genes in this hypoxia cluster have previously been reported to be hypoxia-induced (31 of 45 genes) (references, Table S2), from which we deduced that the remaining genes that have never been reported as such might also be regulated by hypoxia. We chose to examine four genes for hypoxia-induction and cell-type specific expression experiments: triggering receptors expressed by myeloid cells-1 (*TREM-1*), tumor necrosis factor (TNF)-induced protein 3 (*TNFAIP3*), zinc-finger protein 395 (*ZNF395*) and kisspeptin receptor (*KISS1R*).

*TREM1* and *TNFAIP3* (also known as *A20*) are both NF-κB target genes [13], [25]. While *TREM1* is implicated in innate immune response as an amplifier of pro-inflammatory mediators such as IL-8, MCP1 and TNF-α [26], *TNFAIP3* is part of the negative feedback regulatory mechanism of NF-κB and inhibitor of apoptosis [13]. Both genes are known to be expressed in macrophages.

*ZNF395* is a transcription factor that binds to the promoter of the Huntington Disease (*HD*) gene [27]. It's expression has been associated with worse prognosis in Ewing's sarcoma family of tumors (ESFT) [28].

*KISS1R* (also *GPR54*), is a G-coupled protein receptor for the KISS1 peptide, which has been shown to be an inhibitor of tumor metastasis across a range of cancers (reviewed in [29]. KISS1R is known as a regulator of endocrine function and involved in the hypothalamic-pituitary-gonadal axis of the reproductive system [30].

**Figure 4:**
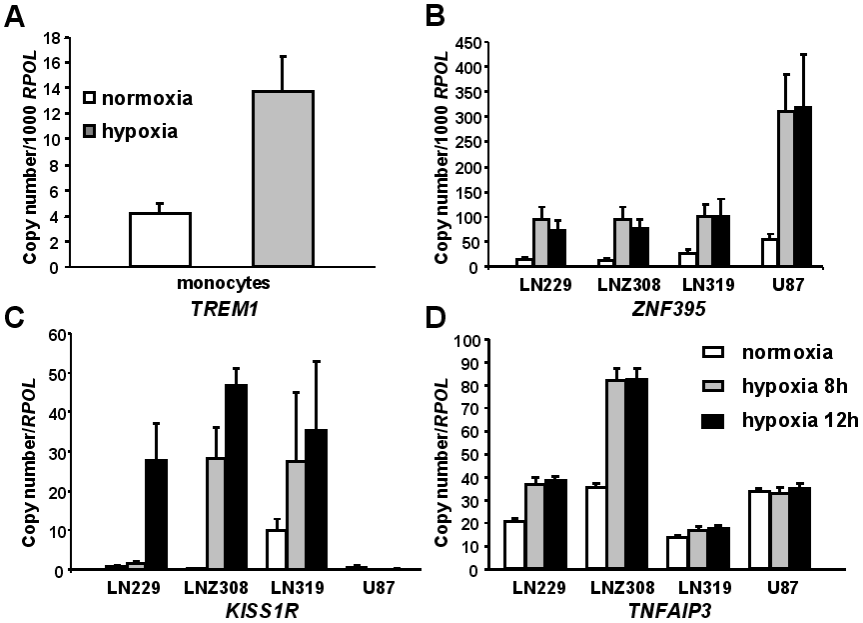
Hypoxia induction of *TREM1, ZNF395* and *KISS1R*. *A*, *TREM1* expression in primary isolated monocytes under normoxia or 18 hours hypoxia (1% O_2_); *B*, *ZNF395*; *C*, *KISS1R* and *D*, *TNFAIP3* expression in four different glioblastoma cell-lines (LN229, LNZ308, LN319, and U87) under normoxia and after 8, and 12 hours of hypoxia (1% O_2_). All results are normalized to expression of the RNA polymerase II (*POLR2A*) gene. Error bars representing standard deviation of triplicate qRT-PCR measurements. Histograms are representative of three independent experiments.

To investigate these genes' hypoxic responsiveness, freshly isolated monocytes and four glioblastoma cell-lines, LN229, LN319, LNZ308 and U87, were cultured under hypoxia (1%O_2_) and the expression of the genes was measured using real-time quantitative RT-PCR (qRT-PCR). Expression of *TREM1* was increased in freshly isolated monocytes after 18 hours of hypoxia as compared to normoxic culture conditions (Fig. 4A). However, neither under normoxia, nor hypoxia *TREM1* expression was detectable in the glioblastoma cell-lines (data not shown), thus supporting the hypothesis that *TREM1* is expressed by inflammatory cells. In contrast, *ZNF395* and *KISS1R* expression and their hypoxia induction were observed in all four glioblastoma cell-lines, while *TNFAIP3* was hypoxia-inducible in LN229 and LNZ308. A time-course revealed that all three genes were induced to a significant level after 8 hours of hypoxia. The levels of expression were either maintained or increased after 12 hours (Fig. 4B–D). In addition, all genes chosen for the hypoxia experiments, except *KISS1R* were also hypoxia-inducible in monocytes. In accordance, assessment of the promoter sequences of *TREM-1, TNFAIP3, ZNF395* and *KISS1R* revealed presence of the consensus HRE A/(G)CGTG. In addition, the *TREM-1* gene promoter comprises a binding sites for the AP-1 transcription factor (TGAGTC/G), *KISS1R*' contains SP-1 binding sites (GGCGGG), while the *ZNF395* and *TNFAIP3* promoters contain both binding sites. AP-1 and SP-1 transcription factors are known to be necessary to form a functional hypoxia transcriptional enhancer complex together with co-activators p300 and CREB Binding Protein (CBP). The genes thus appear to be potentially HIF-1-regulated.

### Expression patterns of *TREM1, ZNF395* and *KISS1R* in glioblastoma

**Figure 5:**
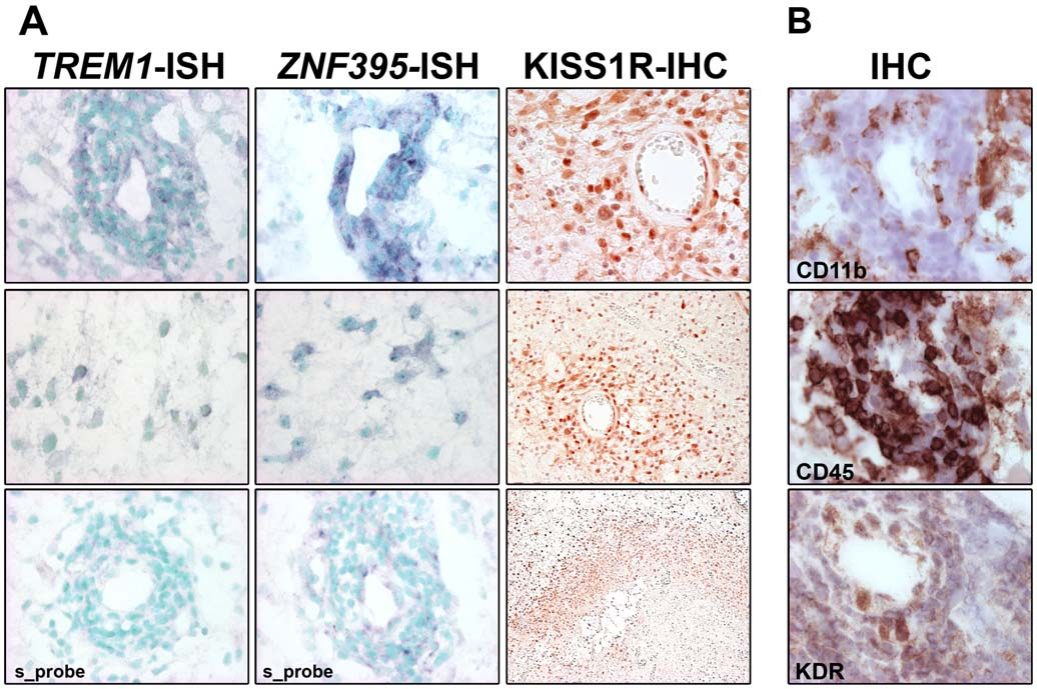
Expression patterns of hypoxia inducible genes in glioblastoma. *A,* In-situ hybridization (ISH) using *TREM1* and *ZNF395* anti-sense probes on sequential frozen sections. Pictures were taken from the same region in proximity to a multilayered blood vessel, and an intermittent region, respectively. A respective sense probe (*s_probe*) was used as negative control. The perinuclear signal for the probes is black-purple (BCIP/NBT), the nuclei are counterstained with methyl green (light blue/green). KISS1R expression was determined by immunohistochemistry (IHC) on paraffin sections: the top panel displays immunoreactivity of the multilayered blood vessel and adjacent cells situated next to a necrosis (original magnification ×40), (middle panel, lower magnification). The bottom panel shows KISSR1 expression in pseudopalisading cells lining a necrosis (original magnification ×10). *B,* IHC against CD11b, a marker for macrophages; CD45, a marker for leukocytes, and KDR that is predominantly expressed by endothelial cells, was performed on sequential sections used for ISH of *TREM1* and *ZNF395* and pictures were taken from the same region close to a multilayered aberrant blood vessel. Original magnifications, ×40.

RNA expression patterns for *TREM1* and *ZNF39*5 were analyzed *in situ* on glioblastoma sections. *TREM1* and *ZNF395* expression were both mostly expressed in proximity of multilayered blood vessels of glioblastoma (Fig. 5). KISS1R immunoreactivity was detected in endothelial cells on blood vessels, as described previously [31] and hypoxic areas adjacent to necrosis, particularly prominent in pseudopalisading cells lining necrosis. For comparison, expression of KDR/VEGFR-2, an angiogenic marker expressed by endothelial cells; CD11b, a monocyte/macrophage marker; and CD45, a pan-leukocyte marker were determined by immunohistochemistry on sequential frozen sections of a glioblastoma used for the *in situ* hybridization (Fig 5).

## Discussion

Investigating a hypoxia-related gene expression signature in glioblastoma we observed two classes of hypoxia-induced genes. The first class, reflecting a common response to hypoxia, includes genes involved in regulating angiogenesis and glycolytic metabolism. The second group comprises genes more commonly expressed in the macrophage/microglia compartment, in accordance with the gene expression profiles of paired samples of glioblastoma and the respective GIM fraction. This second class of genes might reflect the presence of the tumor infiltrating macrophages attracted to hypoxic sites in the tumors. A similar expression pattern was confirmed in independent, published glioblastoma data-sets. Most interestingly, the 2^nd^ PC of the hypoxia cluster was associated with inferior outcome in our dataset of prospectively and homogenously treated glioblastoma patients. A weak trend in the same direction was observed when combining 3 independent external glioblastoma datasets. The weaker signal may be expected given the fact of various treatments, in particular for the chemotherapy component. Our patient cohort was treated homogenously adding an alkylating agent, temozolomide, concomitant and adjuvant to standard radiation, a regimen demonstrated to have improved efficiency.

The importance of the inflammatory response or the involvement of tumor-associated macrophages in tumor promotion have been widely described, but not in the perspective of hypoxia-induced tumor resistance. Although acute hypoxia can induce cell death, exposure to chronic or repeated hypoxia can initiate adaptive changes and select for genetic alterations in tumors that allow survival and proliferation in a hypoxic environment. Our analysis of expression profiles from four independent glioblastoma datasets suggests that hypoxia may induce *TREM1, TNFAIP3* and *BIRC3* in the inflammatory compartment. BIRC3 and TNFAIP3 are known anti-apoptotic factors involved in the NF-κB pathway. Interestingly, both have been implicated in conferring resistance to apoptosis induced by anticancer drugs. BIRC3 is highly expressed in cisplatin-resistant prostate cancer cell lines [32], and in doxorubicine- and busulfan-resistant leukaemia cell lines [33], [34] while TNFAIP3 is overexpressed in tamoxifen-resistant breast carcinoma cell lines [35] and part of a signature for resistance to alkylating agents in glioblastoma cells [36]. Hence, resistance to hypoxia may also trigger resistance to anticancer treatment.

Evidence from clinical and experimental studies have indicated that macrophages can promote tumor progression and metastasis [37]. Macrophages are recruited to the tumor microenvironment by growth factors and chemokines, such as CSF-1, TGF-β, and MCP-1 produced by the tumor cells themselves or cells of the microenvironment, similar to those expressed upon challenge by pathogens or wounding. Once recruited, tumor-educated macrophages take on non-immunological functions, in particular the production of factors that promote progression of tumors to a more malignant state through paracrine cues, including angiogenic factors (VEGF, TNF-α, ANG1), proteases (MMPs), growth factors (FGF, PDGF, TGF-β), and motility factors (EGF, HGF) [3]. Similar to amplifying the immune response, TREM1 may have a role in amplifying these processes in tumors. During innate immune response, engagement of TREM1 stimulates phagocytes to secrete pro-inflammatory cytokines and chemokines, such as IL-8, MCP-1, TNF-α and IL-1 [26]. Interestingly, some of these tumor-promoting factors are also expressed by glioblastoma cells themselves [38], [39], [40]. TREM1 has been shown to be upregulated only in infectious inflammatory responses, but not in non-microbial inflammatory processes [41]. However, in tumors or more likely in tumor-associated macrophages according to our results, *TREM1* may also be induced by hypoxia. High expression of TREM1 in tumor-associated macrophages was reported to correlate with cancer recurrence and poor survival in patients with non-small cell lung cancer [42]. *TREM1* expression in macrophages is also regulated by NF-κB at the transcriptional level [25] again emphasizing the contribution of NF-κB pathway activation in bridging inflammation and tumor promotion and progression. It is known from a number of studies that hypoxia activates NF-κB, and NF-κB has been described to promote malignancy in inflammation-associated cancers by means of its cytoprotective and anti-apoptotic properties [43]. In these cancers, the activation state of NF-κB is controlled by pro-inflammatory mediators produced by neighboring inflammatory cells. In glioblastoma, NF-κB could thus be activated in a similar way, in addition to constitutive NF-κB activity reported from various glioma cell lines and primary cultures from tumor tissue [44]. Of interest is also that *S100A8* and *S100A9* transcripts were found correlated with the inflammatory component in the 2^nd^ PC. S100A8 and S100A9 are chemoattractant proteins that can promote the recruitment of immune-suppressive immune cells into tumor niches [45], and their detection here may therefore be consistent with this notion. In accordance, HIF-1α-mediated recruitment of bone marrow-derived myeloid cells modulating tumor angiogenesis has been shown in mouse models of glioblastoma [4].

Little is known on the potential role of the other two hypoxia inducible genes, *ZNF395* and *KISS1R,* in cancer. Several studies have described a prognostic value of *KISS1* and *KISS1R* expression in tumors across a variety of cancer types and a role in metastasis and trophoblast invasion [46], both invasive processes. Here we showed that KISS1R is expressed in hypoxic areas and endothelial cells of tumor blood vessels, pointing to a putative role in angiogenesis. In accordance, recent evidence proposes a role of kisspeptins in the cardiovascular system, acting as vasoconstrictor with discrete localization of the KISS1R to artherosclerosis-prone vessels [31].

Our results have indicated that hypoxia may induce genes specific to the inflammatory tumor compartment (e.g. macrophages). The relative increase of the hypoxia-inducible and inflammatory-associated genes compared to angiogenesis-related genes was associated with better outcome in our data-set. This is in line with a better outcome associated with the inflammatory response gene cluster (**G24**) as published previously [7] that we show here to be correlated with the inflammatory component of the hypoxia cluster. This observation may seem paradoxical at first since tumor-infiltrating inflammatory cells have been associated with a more malignant phenotype. Likely it is the preponderance of the type of inflammation present – be it either pro-inflammatory consisting of effector anti-tumor responses or anti-inflammatory such as with immune suppressive microglia/macrophages or T regulatory cells that either limit or contribute to tumor malignancy. Alternatively, the inflammatory component may be actually related to its potential to promote tumor vascularization [3], [4] and thereby may improve delivery of the alkylating agent during chemotherapy though increased perfusion. We previously reported that increased expression of a gene signature for blood vessel markers (**G7**) was also associated with better outcome in our patients. This hypothesis with potentially relevant therapeutic implications needs to be validated prospectively in patients treated uniformly with the new standard of care.

Taken together, the investigation of the different components of our hypoxia cluster (loadings of the 2^nd^ PC) and their association with outcome suggest that the effect of hypoxia is more complex than through sole induction of tumor-promoting growth factors or angiogenesis alone. The hypoxia-mediated tumor-host interaction, including the inflammatory compartment may have a profound influence on response to classic therapeutic agents as suggested by our data. Moreover, this hypoxia-modulated interaction may be of clinical relevance for response to anti-angiogenic therapy-mediated induction of hypoxia that has been shown recently to invoke important secondary effects triggering massive tumor invasion [47].

## Materials and Methods

### Ethics Statement

Collection of samples used in this study was approved by the Institutional Review Board of the Faculty of Biology and Medicine of the University of Lausanne.

### Tumor Samples, Gene Expression Profiles, and Patient Characteristics

The microarray data is deposited in the Gene Expression Omnibus (GEO) database at http://www.ncbi.nlm.nih.gov/geo/ (accession-number GSE7696) and described in accordance with MIAME guidelines. In brief, gene expression profiles were established from 80 frozen glioblastoma (grade IV astrocytoma), comprising 70 tumors from initial surgery, and 10 samples resected at recurrence, and from four non-neoplastic brain tissue samples. All glioblastoma patients were treated within two prospective clinical trials that led to establishing combined chemoradiotherapy as the current and worldwide accepted standard of care [10]. All patients provided written informed consent for molecular studies of their tumor. Data from external datasets (Freije, Phillips [8], [9]) were downloaded from GEO, while the Sun data-set [12] was downloaded from the caArray database, publicly accessible at https://caarraydb.nci.nih.gov/caarray/publicExperimentDetailAction.do?expId=101589758985234 (Identifier : gov.nih.nci.ncicb.caarray: Experiment:1015897589852334:1 ). Gene expression data of paired samples of glioblastoma tissue and respective glioma-infiltrating microglia/macrophage (GIM) cell fraction are deposited in GEO (accession-number GSE16119).

### Data Analysis

All statistical analyses were carried out with R, a free software environment available at http://www.r-project.org/ or Coupled Two Way Clustering (CTWC) [48], publicly available at the CTWC-Server: http://ctwc.weizmann.ac.il. The expression intensities for all probe-sets from Affymetrix CEL-files from our and external data sets [7], [8], [9], [12] were estimated using robust multi-array average (RMA) with probe-level quantile normalization followed by median polish summarization as implemented in the BioConductor open source software (available at http://www.bioconductor.org/). Cluster analysis using our data set was performed with CTWC using the expression matrix of 84 samples (80 glioblastoma, 4 non-tumoral brain samples) and 3,860 probe-sets passing a non-specific filter based on standard deviation (>0.75 sd) [7]. All results of CTWC analysis can be viewed at: http://bcf.isb-sib.ch/projects/cancer/glio/ . Probe-sets comprised in stable gene clusters emerging from CTWC served as input for supervised analyses. The genes belonging to the hypoxia cluster were fixed according to the stable hypoxia cluster emerging from CTWC using our dataset (cluster **G84**) [7] (Fig. 1, Table 1). We performed a principal component analysis on the matrix using primary glioblastoma as samples and gene expression values for probe-sets in each mutually exclusive stable cluster as variables. We used the scores of the first and second components to associate samples to survival data. To visualize the contribution of genes to the principal components in Figure 2A, we plotted the loadings i.e. the coefficients of each gene in the linear combination that defines the principal component. The data was scaled and centered independently for each data-set. Univariate or multivariate Cox proportional-hazards models were computed using the mean, the first, and the second principal component (PC) of each mutually exclusive stable cluster. Cox proportional-hazards models were used to examine the association of the hypoxia cluster with survival. For all four data sets, including our own, survival analysis and PC analysis was performed using data from patients at initial surgery (no previous treatment).

### Isolation of Microglia/Macrophages from Human Glioblastoma Tissue

GIM cell fraction was isolated by a modified Percoll-gradient as described previously [24]. Briefly, after resection, freshly isolated glioblastoma tissue was mechanically dissociated through a stainless steel sieve. The dissociated material was centrifuged, washed, and layered onto a Percoll gradient. After centrifugation, the cell layer was removed, washed, layered on top of a second gradient and centrifuged. Microglia/macrophages were collected from the interphase between the 1.065-g/ml and 1.03-g/ml layers.

### Glioblastoma Cell Lines and Human Monocytes

Human glioblastoma cell lines were grown in high glucose Dulbecco's Modified Eagle Medium (DMEM) (Invitrogen) supplemented with 100 units/ml penicillin, 100 µg/ml streptomycin and 5% fetal calf serum (FCS, Bioconcept). Human peripheral blood lymphocytes and monocytes were purified from dextran-sedimented leukocytes. The monocytic and lymphocytic fractions were obtained using the Nycoprep protocol. Cells were maintained under normoxic condition in a humidified incubator, 21% O_2_/5% CO_2_ at 37°C.

### Culture under Hypoxic Condition

Monocytes (1×10^6^) were plated in 3.5 cm tissue culture dishes, incubated under normoxic or hypoxic conditions for 18 hours. For hypoxic conditions, cells were incubated in an Oxoid Gas anaerobic system chamber (Unipath Ltd., Hampshire, UK) using 1% O_2_, 94% N_2_, 5% CO_2_. Glioblastoma cells lines were plated in 10 cm tissue culture dishes. When the cultures reached 80% confluence, fresh culture medium was added and cells were incubated under normoxic or hypoxic conditions as indicated.

### RNA extraction & qRT-PCR

Total RNA was extracted using the Qiagen RNeasy total RNA extraction kit (Qiagen) and reverse transcribed using random hexamers and Superscript RT II (Invitrogen). Quantitative real time PCR was performed in triplicates using the QuantiTect SYBR Green PCR-kit (Qiagen) on a LightCycler 2.0 Instrument (Roche Applied Science). Primer sequences were as follows: TREM-1 (5′-GCAGATAATAAGGGACGGAGA-3′; 5′–CCACTTGGACTGGATGG–3′), ZNF395 (5′–CTGCATGGAAGTCAAAGGAA–3′; 5′–AACCCAATGTCTGAGGGAAC–3′), POLR2A (RefSeq ID NM_000937) (5′–CTGCCAACACAGCCATCTAC–3′; 5′–TCACCCATTCCTGATCCTCT–3′) and TNFAIP3 as previously published [36]. Quantitect Primers (Qiagen) were used for KISS1R. The following PCR conditions were used: 95°C, 15 minutes; 45 cycles at 94°C, 30 seconds; 50°C, 30 seconds; 72°C, 20 seconds. The measured transcript abundance was normalized to the level of POLR2A (RNA Polymerase II) for all samples.

### *In situ* Hybridization and Immunohistochemistry

Fragments of the *TREM-1* and *ZNF395* cDNAs were obtained by PCR using specific primers (*TREM-1*: forward 5′-TTGTCTCAGAACTCCGAGCTGC-3′, reverse 5′-GAGACATCGGCAGTTGACTTGG-3′; *ZNF395*: forward 5′-TTTTGGTTCTCCCCAAACTG-3, reverse 5′-GGTGGAAGAGCAGACAGAGG-3′). PCR products were purified and cloned into pBluescript-KS-M13+. Digoxigenin-11-UTP (DIG)-labeled riboprobes were synthesized from *×ba*I or *Pst*I linearized plasmids by *in vitro* transcription reaction with T7 or Sp6 polymerase and with incorporation of DIG, and used for *in situ* hybridization as described (protocol Roche Applied Science). Briefly, freshly cut frozen sections were fixed with 4% paraformaldehyde and hybridized with 0.4 ng/µl riboprobes in hybridization buffer (50% formamide, 4× SSC, 10% dextran sulfate, 1× Denhardts solution, 2 mM EDTA, and 500 µg/ml sheared salmon sperm DNA) at 45°C overnight, followed by increasingly stringent washes of SSC to 0.02×, immunodetection using anti-DIG antibody (1∶200 dilution), and stained by a combination of BCIP (5-Bromo-4-Chloro-3′-Indolyphosphate p-Toluidine Salt) and NBT (Nitro-Blue Tetrazolium Chloride) (Roche Applied Science). Slides were subsequently counterstained with methyl green.

Immunohistochemistry for CD11b (Pharmingen, dilution 1∶100), CD45 (DAKO, dilution 1∶100) and KDR (Santa Cruz, dilution 1∶500) were performed on 5 µm frozen sections according to standard procedures using 5 min cold acetone fixation and 1 hour incubation with the primary antibody. Expresssion of KISS1R was evaluated on paraffin embedded glioblastoma samples (NOVUS Biologicals, GPR54 antibody, dilution 1∶500) according to standard procedures using high temperature epitope retrieval (citrate buffer pH 6.0, pressure cooker 20 min).

## Supporting Information

Table S1 (0.06 MB DOC)

Table S2 (0.12 MB DOC)
