# Supplementary material for: Modulation of Angiogenic and Inflammatory Response in Glioblastoma by Hypoxia
Source: PLoS One. 2009 Jun 17;4(6):e5947. doi: 10.1371/journal.pone.0005947 (PMC2694268; doi:10.1371/journal.pone.0005947)
Supplement: Table S1 — (0.06 MB DOC) [file pone.0005947.s001.doc]

**Table S1. Top Anti-correlated Genes to the 2^nd^ PC***

| ProbeSet | Gene Symbol | Gene Title | G24^#^ |
| --- | --- | --- | --- |
| 1552807_a_at | SIGLEC10 | sialic acid binding Ig-like lectin 10 | 1 |
| 1554899_s_at | FCER1G | Fc fragment of IgE, high affinity I, receptor for; gamma polypeptide | 1 |
| 1555349_a_at | ITGB2 | integrin, beta 2 (antigen CD18 (p95), lymphocyte function-associated antigen 1; macrophage antigen 1 (mac-1) beta subunit) | 1 |
| 1555728_a_at | MS4A4A | membrane-spanning 4-domains, subfamily A, member 4 | 1 |
| 201422_at | IFI30 | interferon, gamma-inducible protein 30 /// interferon, gamma-inducible protein 30 | 1 |
| 201721_s_at | LAPTM5 | Lysosomal-associated multispanning membrane protein-5 /// Lysosomal-associated multispanning membrane protein-5 | 1 |
| 201943_s_at | CPD | carboxypeptidase D |  |
| 202075_s_at | PLTP | phospholipid transfer protein /// phospholipid transfer protein |  |
| 202546_at | VAMP8 | vesicle-associated membrane protein 8 (endobrevin) /// vesicle-associated membrane protein 8 (endobrevin) | 1 |
| 202803_s_at | ITGB2 | integrin, beta 2 (antigen CD18 (p95), lymphocyte function-associated antigen 1; macrophage antigen 1 (mac-1) beta subunit) /// integrin, beta 2 (antigen CD18 (p95), lymphocyte function-associated antigen 1; macrophage antigen 1 (mac-1) beta subunit) | 1 |
| 202833_s_at | SERPINA1 | serine (or cysteine) proteinase inhibitor, clade A (alpha-1 antiproteinase, antitrypsin), member 1 | 1 |
| 202917_s_at | S100A8 | S100 calcium binding protein A8 (calgranulin A) /// S100 calcium binding protein A8 (calgranulin A) | 1 |
| 202957_at | HCLS1 | hematopoietic cell-specific Lyn substrate 1 /// hematopoietic cell-specific Lyn substrate 1 | 1 |
| 203305_at | F13A1 | coagulation factor XIII, A1 polypeptide /// coagulation factor XIII, A1 polypeptide | 1 |
| 203416_at | CD53 | CD53 antigen /// CD53 antigen | 1 |
| 203535_at | S100A9 | S100 calcium binding protein A9 (calgranulin B) /// S100 calcium binding protein A9 (calgranulin B) | 1 |
| 203691_at | PI3 | protease inhibitor 3, skin-derived (SKALP) |  |
| 204174_at | ALOX5AP | arachidonate 5-lipoxygenase-activating protein /// arachidonate 5-lipoxygenase-activating protein | 1 |
| 204232_at | FCER1G | Fc fragment of IgE, high affinity I, receptor for; gamma polypeptide /// Fc fragment of IgE, high affinity I, receptor for; gamma polypeptide | 1 |
| 204446_s_at | ALOX5 | arachidonate 5-lipoxygenase /// arachidonate 5-lipoxygenase | 1 |
| 204563_at | SELL | selectin L (lymphocyte adhesion molecule 1) /// selectin L (lymphocyte adhesion molecule 1) | 1 |
| 204787_at | Z39IG | Ig superfamily protein /// Ig superfamily protein | 1 |
| 204959_at | MNDA | myeloid cell nuclear differentiation antigen /// myeloid cell nuclear differentiation antigen | 1 |
| 205027_s_at | MAP3K8 | mitogen-activated protein kinase kinase kinase 8 /// mitogen-activated protein kinase kinase kinase 8 | 1 |
| 205119_s_at | FPR1 | formyl peptide receptor 1 /// formyl peptide receptor 1 | 1 |
| 205476_at | CCL20 | chemokine (C-C motif) ligand 20 /// chemokine (C-C motif) ligand 20 |  |
| 205681_at | BCL2A1 | BCL2-related protein A1 /// BCL2-related protein A1 | 1 |
| 206111_at | RNASE2 | ribonuclease, RNase A family, 2 (liver, eosinophil-derived neurotoxin) /// ribonuclease, RNase A family, 2 (liver, eosinophil-derived neurotoxin) | 1 |
| 206392_s_at | RARRES1 | retinoic acid receptor responder (tazarotene induced) 1 |  |
| 206584_at | LY96 | lymphocyte antigen 96 | 1 |
| 208018_s_at | HCK | hemopoietic cell kinase /// hemopoietic cell kinase | 1 |
| 210176_at | TLR1 | toll-like receptor 1 /// toll-like receptor 1 | 1 |
| 211429_s_at | --- | PRO2275 mRNA, complete cds /// PRO2275 mRNA, complete cds | 1 |
| 212268_at | SERPINB1 | serine (or cysteine) proteinase inhibitor, clade B (ovalbumin), member 1 |  |
| 214974_x_at | CXCL5 | chemokine (C-X-C motif) ligand 5 |  |
| 216598_s_at | CCL2 | chemokine (C-C motif) ligand 2 /// chemokine (C-C motif) ligand 2 |  |
| 217388_s_at | KYNU | kynureninase (L-kynurenine hydrolase) | 1 |
| 218232_at | C1QA | complement component 1, q subcomponent, alpha polypeptide /// complement component 1, q subcomponent, alpha polypeptide | 1 |
| 220491_at | HAMP | hepcidin antimicrobial peptide /// hepcidin antimicrobial peptide | 1 |
| 221698_s_at | CLECSF12 | C-type (calcium dependent, carbohydrate-recognition domain) lectin, superfamily member 12 /// C-type (calcium dependent, carbohydrate-recognition domain) lectin, superfamily member 12 | 1 |
| 224356_x_at | MS4A6A | membrane-spanning 4-domains, subfamily A, member 6A /// membrane-spanning 4-domains, subfamily A, member 6A | 1 |
| 226142_at | HRB2 | HIV-1 rev binding protein 2 |  |
| 226218_at | IL7R | interleukin 7 receptor |  |
| 227961_at | CTSB | cathepsin B |  |
| 232231_at | --- | MRNA; cDNA DKFZp761J1112 (from clone DKFZp761J1112) |  |
| 41469_at | PI3 | protease inhibitor 3, skin-derived (SKALP) |  |

* >0.5 Pearson Correlation

^#^ Probesets part of Immune/inflammatory Response Cluster G24 identified by CTWC [1]
