# Supplementary material for: Modulation of Angiogenic and Inflammatory Response in Glioblastoma by Hypoxia
Source: PLoS One. 2009 Jun 17;4(6):e5947. doi: 10.1371/journal.pone.0005947 (PMC2694268; doi:10.1371/journal.pone.0005947)
Supplement: Table S2 — (0.12 MB DOC) [file pone.0005947.s002.doc]

Table S2. References of Hypoxia-induced Gene Cluster *

| Gene Title | Gene Symbol |  | References |
| --- | --- | --- | --- |
| basic helix-loop-helix domain containing, class B, 2 | BHLHB2 |  | [2] |
| procollagen-proline, 2-oxoglutarate 4-dioxygenase (proline 4-hydroxylase), alpha polypeptide II | P4HA2 |  | [3] |
| caldesmon 1 | CALD1 |  |  |
| carbonic anhydrase XII | CA12 |  | [4] |
| tumor necrosis factor, alpha-induced protein 3 | TNFAIP3 |  | This work |
| tumor necrosis factor, alpha-induced protein 3 | TNFAIP3 |  | This work |
| chemokine (C-X-C motif) ligand 3 | CXCL3 |  |  |
| serpin peptidase inhibitor, clade E (nexin, plasminogen activator inhibitor type 1), member 1 | SERPINE1 |  | [5] |
| N-myc downstream regulated gene 1 | NDRG1 |  | [6] |
| solute carrier family 2 (facilitated glucose transporter), member 3 | SLC2A3 |  | [7] |
| interleukin 8 | IL8 |  | [8] |
| G protein-coupled receptor, family C, group 5, member A | GPRC5A |  | [9,10] |
| phosphoglycerate kinase 1 | PGK1 |  | [11] |
| adipose differentiation-related protein | ADFP |  | [12] |
| hypoxia-inducible protein 2 | HIG2 |  | [13] |
| adrenomedullin | ADM |  | [14,15] |
| angiopoietin-like 4 | ANGPTL4 |  | [16] |
| adenylosuccinate synthase like 1 | ADSSL1 |  |  |
| lysyl oxidase | LOX |  | [17] |
| lysyl oxidase | LOX |  | [17] |
| hexokinase 2 | HK2 |  | [18] |
| vascular endothelial growth factor A | VEGFA |  | [19] |
| vascular endothelial growth factor A | VEGFA |  | [19] |
| vascular endothelial growth factor A | VEGFA |  | [19] |
| basic helix-loop-helix domain containing, class B, 2 | BHLHB2 |  | [2] |
| vascular endothelial growth factor A | VEGFA |  | [19] |
| pyruvate dehydrogenase kinase, isozyme 1 | PDK1 |  | [20] |
| glucan (1,4-alpha-), branching enzyme 1 (glycogen branching enzyme, Andersen disease, glycogen storage disease type IV) | GBE1 |  | [21] |
| arrestin domain containing 3 | ARRDC3 |  |  |
| zinc finger protein 395 | ZNF395 |  | This work |
| ERO1-like (S. cerevisiae) | ERO1L |  | [22] |
| KISS1 receptor | KISS1R |  | This work |
| enolase 2 (gamma, neuronal) | ENO2 |  | [23] |
| zinc finger protein 395 | ZNF395 |  | This work |
| stanniocalcin 2 | STC2 |  | [24] |
| zinc finger protein 395 /// F-box protein 16 | FBXO16 /// ZNF395 |  | This work |
| zinc finger protein 395 /// F-box protein 16 | FBXO16 /// ZNF395 |  | This work |
| transmembrane protein 45A | TMEM45A |  |  |
| v-maf musculoaponeurotic fibrosarcoma oncogene homolog F (avian) | MAFF |  | [25] |
| solute carrier family 39 (zinc transporter), member 14 | SLC39A14 |  |  |
| solute carrier family 2 (facilitated glucose transporter), member 3 | SLC2A3 |  | [7] |
| solute carrier family 2 (facilitated glucose transporter), member 3 /// solute carrier family 2 (facilitated glucose transporter), member 14 | SLC2A14 /// SLC2A3 |  | [7] |
| neuritin 1 | NRN1 |  | [26] |
| family with sequence similarity 20, member C | FAM20C |  |  |
| heat shock 70kDa protein 6 (HSP70B') | HSPA6 |  |  |
| triggering receptor expressed on myeloid cells 1 | TREM1 |  | This work, [27] |
| stanniocalcin 1 | STC1 |  | [28] |
| Stanniocalcin 1 | STC1 |  | [28] |
| solute carrier family 16, member 10 (aromatic amino acid transporter) | SLC16A10 |  | [17] |
| Transcribed locus | --- |  |  |
| baculoviral IAP repeat-containing 3 | BIRC3 |  | [29] |
| chromosome 15 open reading frame 48 | C15orf48 |  |  |

*Probesets of Hypoxia Cluster G84 defined by CTWC [1]

## References of the Supporting Information

## 1. Murat A, Migliavacca E, Gorlia T, Lambiv WL, Shay T, et al. (2008) Stem cell-related "self-renewal" signature and high epidermal growth factor receptor expression associated with resistance to concomitant chemoradiotherapy in glioblastoma. J Clin Oncol 26: 3015-3024.

## 2. St-Pierre B, Flock G, Zacksenhaus E, Egan SE (2002) Stra13 homodimers repress transcription through class B E-box elements. J Biol Chem 277: 46544-46551.

## 3. Hofbauer KH, Gess B, Lohaus C, Meyer HE, Katschinski D, et al. (2003) Oxygen tension regulates the expression of a group of procollagen hydroxylases. Eur J Biochem 270: 4515-4522.

## 4. Wykoff CC, Beasley NJ, Watson PH, Turner KJ, Pastorek J, et al. (2000) Hypoxia-inducible expression of tumor-associated carbonic anhydrases. Cancer Res 60: 7075-7083.

## 5. Pinsky DJ, Liao H, Lawson CA, Yan SF, Chen J, et al. (1998) Coordinated induction of plasminogen activator inhibitor-1 (PAI-1) and inhibition of plasminogen activator gene expression by hypoxia promotes pulmonary vascular fibrin deposition. J Clin Invest 102: 919-928.

## 6. Caruso RP, Levinson B, Melamed J, Wieczorek R, Taneja S, et al. (2004) Altered N-myc downstream-regulated gene 1 protein expression in African-American compared with caucasian prostate cancer patients. Clin Cancer Res 10: 222-227.

## 7. Ebert BL, Gleadle JM, O'Rourke JF, Bartlett SM, Poulton J, et al. (1996) Isoenzyme-specific regulation of genes involved in energy metabolism by hypoxia: similarities with the regulation of erythropoietin. Biochem J 313 ( Pt 3): 809-814.

## 8. Metinko AP, Kunkel SL, Standiford TJ, Strieter RM (1992) Anoxia-hyperoxia induces monocyte-derived interleukin-8. J Clin Invest 90: 791-798.

## 9. Kambe T, Tada-Kambe J, Kuge Y, Yamaguchi-Iwai Y, Nagao M, et al. (2000) Retinoic acid stimulates erythropoietin gene transcription in embryonal carcinoma cells through the direct repeat of a steroid/thyroid hormone receptor response element half-site in the hypoxia-response enhancer. Blood 96: 3265-3271.

## 10. Tao Q, Cheng Y, Clifford J, Lotan R (2004) Characterization of the murine orphan G-protein-coupled receptor gene Rai3 and its regulation by retinoic acid. Genomics 83: 270-280.

## 11. Salceda S, Beck I, Caro J (1996) Absolute requirement of aryl hydrocarbon receptor nuclear translocator protein for gene activation by hypoxia. Arch Biochem Biophys 334: 389-394.

## 12. Saarikoski ST, Rivera SP, Hankinson O (2002) Mitogen-inducible gene 6 (MIG-6), adipophilin and tuftelin are inducible by hypoxia. FEBS Lett 530: 186-190.

## 13. Denko N, Schindler C, Koong A, Laderoute K, Green C, et al. (2000) Epigenetic regulation of gene expression in cervical cancer cells by the tumor microenvironment. Clin Cancer Res 6: 480-487.

## 14. Nakayama M, Takahashi K, Murakami O, Shirato K, Shibahara S (1998) Induction of adrenomedullin by hypoxia and cobalt chloride in human colorectal carcinoma cells. Biochem Biophys Res Commun 243: 514-517.

## 15. Oehler MK, Norbury C, Hague S, Rees MC, Bicknell R (2001) Adrenomedullin inhibits hypoxic cell death by upregulation of Bcl-2 in endometrial cancer cells: a possible promotion mechanism for tumour growth. Oncogene 20: 2937-2945.

## 16. Belanger AJ, Lu H, Date T, Liu LX, Vincent KA, et al. (2002) Hypoxia up-regulates expression of peroxisome proliferator-activated receptor gamma angiopoietin-related gene (PGAR) in cardiomyocytes: role of hypoxia inducible factor 1alpha. J Mol Cell Cardiol 34: 765-774.

## 17. Denko NC, Fontana LA, Hudson KM, Sutphin PD, Raychaudhuri S, et al. (2003) Investigating hypoxic tumor physiology through gene expression patterns. Oncogene 22: 5907-5914.

## 18. Riddle SR, Ahmad A, Ahmad S, Deeb SS, Malkki M, et al. (2000) Hypoxia induces hexokinase II gene expression in human lung cell line A549. Am J Physiol Lung Cell Mol Physiol 278: L407-416.

## 19. Shweiki D, Itin A, Soffer D, Keshet E (1992) Vascular endothelial growth factor induced by hypoxia may mediate hypoxia-initiated angiogenesis. Nature 359: 843-845.

## 20. Kim JW, Tchernyshyov I, Semenza GL, Dang CV (2006) HIF-1-mediated expression of pyruvate dehydrogenase kinase: a metabolic switch required for cellular adaptation to hypoxia. Cell Metab 3: 177-185.

## 21. Zhao J, Chen H, Davidson T, Kluz T, Zhang Q, et al. (2004) Nickel-induced 1,4-alpha-glucan branching enzyme 1 up-regulation via the hypoxic signaling pathway. Toxicol Appl Pharmacol 196: 404-409.

## 22. Gess B, Hofbauer KH, Wenger RH, Lohaus C, Meyer HE, et al. (2003) The cellular oxygen tension regulates expression of the endoplasmic oxidoreductase ERO1-Lalpha. Eur J Biochem 270: 2228-2235.

## 23. Olbryt M, Jarzab M, Jazowiecka-Rakus J, Simek K, Szala S, et al. (2006) Gene expression profile of B 16(F10) murine melanoma cells exposed to hypoxic conditions in vitro. Gene Expr 13: 191-203.

## 24. Leonard MO, Cottell DC, Godson C, Brady HR, Taylor CT (2003) The role of HIF-1 alpha in transcriptional regulation of the proximal tubular epithelial cell response to hypoxia. J Biol Chem 278: 40296-40304.

## 25. Chen L, Fink T, Ebbesen P, Zachar V (2006) Temporal transcriptome of mouse ATDC5 chondroprogenitors differentiating under hypoxic conditions. Exp Cell Res 312: 1727-1744.

## 26. Le Jan S, Le Meur N, Cazes A, Philippe J, Le Cunff M, et al. (2006) Characterization of the expression of the hypoxia-induced genes neuritin, TXNIP and IGFBP3 in cancer. FEBS Lett 580: 3395-3400.

## 27. Bosco MC, Puppo M, Santangelo C, Anfosso L, Pfeffer U, et al. (2006) Hypoxia modifies the transcriptome of primary human monocytes: modulation of novel immune-related genes and identification of CC-chemokine ligand 20 as a new hypoxia-inducible gene. J Immunol 177: 1941-1955.

## 28. Lal A, Peters H, St Croix B, Haroon ZA, Dewhirst MW, et al. (2001) Transcriptional response to hypoxia in human tumors. J Natl Cancer Inst 93: 1337-1343.

## 29. Dong Z, Venkatachalam MA, Wang J, Patel Y, Saikumar P, et al. (2001) Up-regulation of apoptosis inhibitory protein IAP-2 by hypoxia. Hif-1-independent mechanisms. J Biol Chem 276: 18702-18709.
